# Antibiotic use in rural Kenyan livestock: navigating misuse, experience gaps and AMR risks

**DOI:** 10.1099/mic.0.001582

**Published:** 2025-07-11

**Authors:** Lucy Obolensky, Esbon Wambugu, Edna K. Kubai, Iain Doig, Miriam Beattie, Michael J. Dillon

**Affiliations:** 1 Peninsula Medical School, University of Plymouth, Plymouth, PL4 8AA, UK; 2 County Government of Laikipia, Department of Health, Nanyuki, Kenya; 3 Bristol Royal Infirmary, Bristol, BS2 8HW, UK; 4 Royal Cornwall Hospital, Truro, TR1 3LJ, UK

**Keywords:** antimicrobial resistance (AMR), global health, Kenya, One Health, pastoral

## Abstract

Antimicrobial resistance (AMR) is an escalating global health threat, with the greatest risk observed in low- to middle-income countries, particularly in the global south. The World Health Organization advocates for a One Health approach to address AMR, promoting collaboration across sectors, including in agriculture. This study aims to enhance understanding of antimicrobial use and stewardship in livestock within pastoralist communities in northern Kenya, where there is limited information. The study employed a qualitative approach, using semi-structured interviews to gather data on farming practices and antibiotic use. Interviews were conducted by trained volunteers proficient in Swahili and Ma (a Maasai language), across four pastoralist communities in northern Kenya in December 2023. The data were then thematically analysed by four researchers. Fifty-one individuals participated in the study. Thematic analysis revealed several key insights, including the widespread misuse of antibiotics, often used on intuition and without professional support. A notable barrier to appropriate use was the lack of veterinary advice, with many participants relying on agrovets or past experience for guidance. Cross-use of antibiotics, such as administering animal antibiotics to humans, was also observed. Awareness of AMR was limited, and leftover antibiotics were often saved or shared across communities. The findings from this study underscore the critical need for targeted education and training within these communities.

**Impact Statement**

This study sheds light on antibiotic use practices and knowledge of antimicrobial resistance (AMR) amongst livestock farmers in rural Kenya. It provides important insights into how antibiotics are applied in the treatment of animals and highlights significant gaps in understanding, particularly regarding the distinction between antibiotics and anthelmintics. The findings demonstrate the widespread, unregulated use of antibiotics, including their use in humans and animals interchangeably, and a lack of consistent dosing practices. This contributes to the growing body of evidence on antibiotic misuse in low- and middle-income countries (LMICs), where access to veterinary services is often limited, and awareness of AMR remains low.

The study is relevant to a wide audience, including policymakers, veterinarians, public health officials and researchers focused on tackling AMR. By documenting the fluid and informal use of antibiotics in rural communities, it provides valuable evidence to inform targeted interventions that can improve antibiotic stewardship in livestock farming.

The significance of this research lies in its potential to inform strategies that reduce antibiotic misuse, thereby slowing the development of AMR. This study adds incrementally to the literature, emphasizing the urgent need for education and accessible veterinary services to mitigate the risks associated with inappropriate antibiotic practices in LMICs.

## Data Availability

The questionnaire and supplementary datasets are available from PEARL, an open-access repository (https://doi.org/10.24382/3b8ddedd-d6d0-4c2d-8b7a-2d4fea7a9ee3) [1].

## Introduction

The use of antibiotics in livestock management is a common practice worldwide, facilitating disease control and promoting productivity. However, the inappropriate administration of these drugs has raised significant public health concerns due to its contribution to antimicrobial resistance (AMR) [2,3]. AMR occurs when a pathogen develops the ability to resist treatments that were previously effective; it threatens to undermine global healthcare systems and underscores an urgent need for responsible antibiotic stewardship, particularly within the agricultural sector [4]. This issue is especially pronounced in low- and middle-income countries (LMICs), where access to veterinary services may be limited, and regulatory oversight is often minimal [5].

In many LMICs, rural communities rely heavily on livestock for economic stability, subsistence and social identity [6], and within pastoralist societies, livestock farming serves as the primary livelihood for millions of people [7]. However, pastoralist communities face systemic limitations across several interlinked Sustainable Development Goals (SDGs), including SDG 1 (no poverty), SDG 2 (zero hunger), SDG 3 (good health and well-being) and SDG 13 (climate action) [8]. For example, climate change is driving prolonged droughts, unpredictable rainfall patterns and intensified heat stress, all of which reduce the availability of water and pasturelands crucial for grazing [9]. Additionally, the rapid depletion of rangelands due to population pressure, land fragmentation and overgrazing has left pastoralists increasingly vulnerable to food insecurity and economic instability [9]. Consequently, in the arid and semi-arid lands of Kenya, including regions such as Laikipia and Isiolo, these challenges necessitate adaptive and resilient livestock practices.

Pastoralists in these regions can face high livestock morbidity and mortality rates due to disease, leading to liberal antibiotic use as preventive and curative measures [10]. Whilst antibiotics are critical for sustaining livestock health in these vulnerable settings, these increasing pressures have fostered informal, often unregulated antibiotic practices [11,12]. A lack of veterinary infrastructure [13], combined with the unregulated sale of antibiotics through agrovet shops and general stores, has facilitated a culture of self-administered treatments amongst livestock owners. These trends can lead to suboptimal dosing, incomplete treatment courses and unsanctioned antibiotic sharing between human and animal populations, each exacerbating the risk of AMR [14,15].

The potential for antibiotics to leave residual compounds in animal products further elevates the public health risks associated with AMR. Studies on antibiotic residues in animal-derived foods have shown that consumption shortly after antibiotic administration can expose humans to trace antibiotic levels, potentially facilitating resistance in humans [16,18].

This study aims to explore antibiotic usage patterns, perceptions and practices amongst rural livestock farmers in northern Kenya, particularly the counties of Laikipia and Isiolo, in the areas of Leparua, Mutunyi, Naibor and Ngare Ndare. Similar qualitative research has been conducted in Kenya and Eastern Africa, particularly in relation to poultry and dairy farming; however, there remains limited data on antibiotic use within pastoralist communities, especially in the Laikipia and Isiolo [13,19,21]. By examining the experiences, attitudes and current beliefs of these farmers, the research provides insights into the local drivers of antibiotic misuse and the consequent AMR risk.

## Methods

### Study design

The interview questionnaire was developed collaboratively between Kenyan researchers, UK-based researchers and community members and local farmers in Leparua.

Researchers and community members co-created the questionnaire through a series of discussions and iterative conversations, shaping its content to reflect the lived experiences and linguistic norms of the target participants. This ensured that the questionnaire maintained cultural appropriateness and relevance to the local context.

The collaborative design process served as an informal validation, with farmers and community representatives in Leparua actively refining the questionnaire in a live pre-test setting. This made a separate formal pilot study unnecessary.

### Participant recruitment

The research was conducted across four locations in northern Kenya: Leparua, Mutunyi, Naibor and Ngare Ndare. These sites were selected due to existing relationships with the research team and their proximity to the Kenyan research team, allowing for regular access and engagement. In each location, farmers play a central role in the community and are well known locally, making word of mouth a practical and effective recruitment approach.

Participants were selected using snowball sampling, beginning with individuals known to the research team and local contacts. These initial participants recommended others involved in agriculture. Eligible participants were aged 18 or older, with no restrictions based on gender or level of farming experience. This approach helped reach a diverse range of farmers embedded in their local contexts.

Interviews took place at communal gathering points in each village. Participants were not paid for completing the questionnaire but were provided with financial compensation to cover their travel expenses to and from the interview site.

A formal sample size was not calculated in advance, as the study employed a qualitative, exploratory approach with a focus on local relevance and community engagement. Participant recruitment was shaped by accessibility and willingness to participate, rather than statistical representativeness. That said, efforts were made to ensure diversity in age, gender and farming experience amongst those who did participate.

### Data collection

The interviews were conducted in person, by a team of local researchers who travelled to the four sites over the course of 1 week.

Initially, group interviews were planned; however, during the initial phase, it became clear that cultural norms might limit the participation of younger individuals in group settings, as they appeared to defer to older participants. In light of this, the study design was adapted to employ semi-structured, one-on-one interviews, allowing for more equitable participation across age groups.

To ease participants into the interviews and build rapport, participants were initially asked, ‘Do you ever give your livestock antibiotics?’ This did not specify a reference period and was designed to be open-ended to set the tone for the subsequent, more detailed questions.

The questionnaire was written in English; however, most participants were unlikely to speak English. Therefore, the questionnaire was verbally translated by the research team during the interviews into Swahili, Ma or other local dialects as necessary. Responses were then translated and recorded in English by the team, in real time, during the interviews.

### Data analysis

A reflexive thematic analysis was conducted, using a collaborative, pragmatic lens [22]. Two members of the project team independently coded the interview transcripts using a combination of inductive and deductive approaches. The researchers then met to discuss their coding, compare interpretations and refine the initial coding matrix. A third researcher then independently applied the agreed-upon coding framework to the full dataset to check for consistency and completeness. There were no major disagreements during this phase, though some variations emerged in the emphasis placed on certain themes due to nuanced interpretations. These were resolved through open discussion and input from the Kenyan research team, who ensured that the cultural and contextual relevance was accurately represented.

Microsoft Excel was used to collate coded extracts, track theme development and calculate descriptive statistics such as frequencies and percentages. Key themes relating to antibiotic use, procurement practices and awareness of AMR were identified.

Antibiotic use practices included patterns and reasons for antibiotic use in livestock, such as symptom recognition, prophylactic use and growth promotion.

Procurement practices and sources of advice encompassed how participants obtained antibiotics and sought guidance on their use, including reliance on agrovets, leaflets, veterinary services and informal sources.

Awareness and understanding of AMR focused on participants’ knowledge, perceptions and practices related to AMR, including misconceptions about antibiotics and their distinction from other drug classes.

## Results

### Participant recruitment

**Fig. 1.**
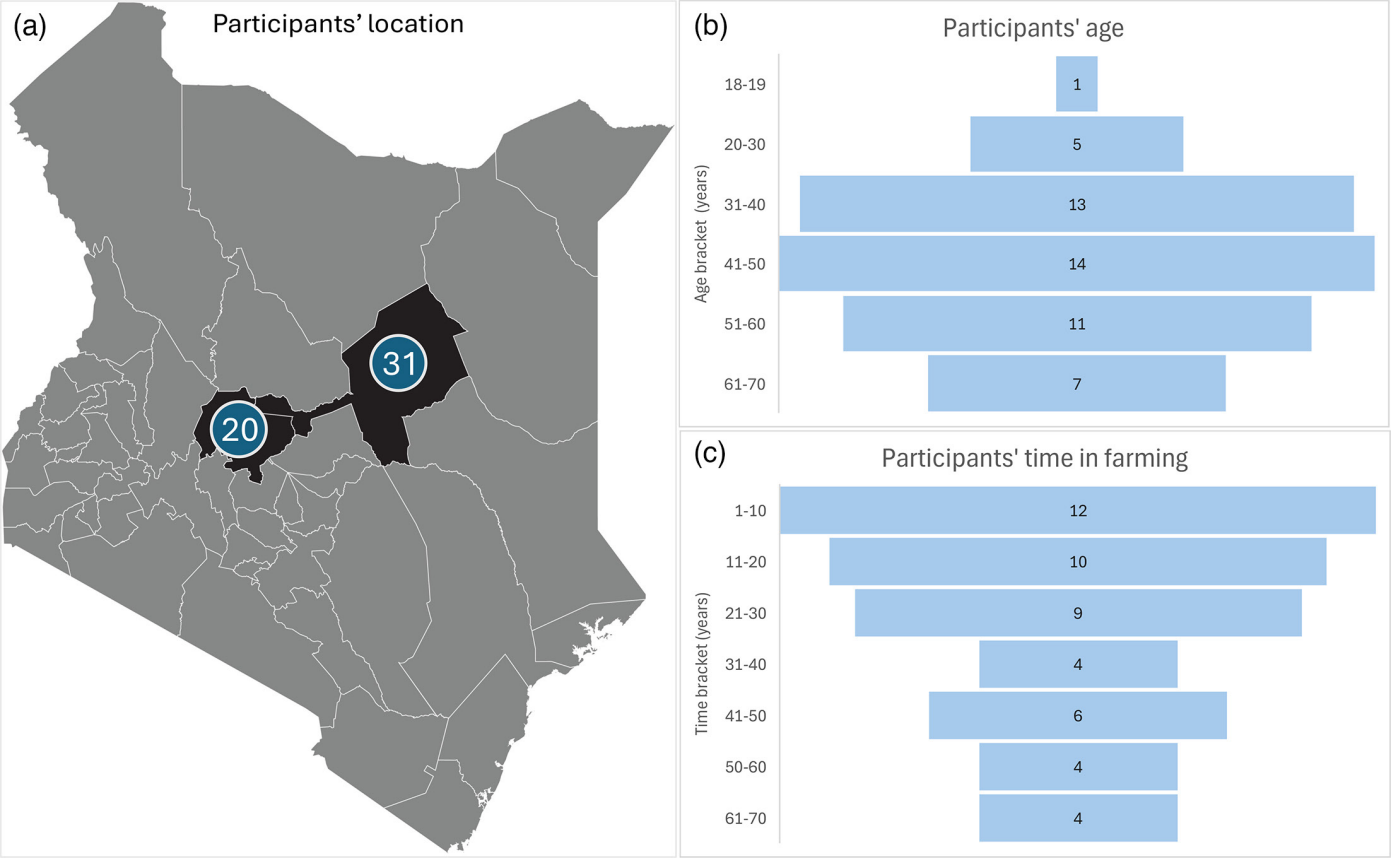
Characteristics of study participants. (**a**) Map of Kenya indicating the geographical locations of study participants. There were 20 participants from Laikipia County, specifically Leparua and Mutunyi. There were 31 participants from Isiolo County, specifically Naibor and Ngare Ndare. All participants came from similar cultural backgrounds. (**b**) Age distribution of participants, showing the proportion across different age groups. (**c**) Farming experience of participants, represented in years. Map adapted from Momcilo/stock.adobe.com.

A total of 51 participants were interviewed from four areas: Leparua, Mutunyi, Naibor and Ngare Ndare (Fig. 1a). Amongst the participants, 31% (*n*=16) were female, and 69% (*n*=35) were male, with a mean age of 45 years (Fig. 1b). On average, participants had spent 27.2 years working as pastoralists, indicating significant experience in livestock management (Fig. 1c).

### Livestock ownership and income sources

Participants owned a variety of livestock, primarily goats, sheep, cows and chickens, with most operating subsistence-oriented farms largely focused on day-to-day survival and self-reliance. The size of these farms was reflected in the number of animals owned, typically fewer than 1,000 chickens or 500 goats. Whilst all participants cited livestock as their main source of income, several also engaged in other activities to supplement their earnings, including crop production, beekeeping, providing transport services using motorbikes and casual labour.

### Criteria for administering antibiotics

As expected, all participants (100%) reported using antibiotics for their livestock when animals appeared ill. However, less than half (45%) of respondents specified observable symptoms that guided their decision to administer antibiotics, with 22% (*n*=11) citing coughing, 16% (*n*=8) identifying poor feeding and 10% (*n*=5) mentioning diarrhoea. Notably, 6% (*n*=3) mentioned using antibiotics to increase an animal’s weight or milk production, and one participant (2%) reported administering metaprophylactic antibiotics to the entire herd if a single animal became ill or died, reflecting a significant variation in antibiotic usage strategies.

### Types of antibiotics used

Oxytetracycline was the most frequently reported active ingredient, used by 86% of participants (*n*=44), with the most commonly cited brand being referred to as ‘Adamycin’ (Table 1). Penicillin was the second most frequently used antibiotic (43%, *n*=22), followed by tetracycline (12%, *n*=6) and tylosin (10%, *n*=5). Other antibiotics, such as amoxicillin and tobramycin, were each reported by 2% of participants (*n*=1 respectively).

**Table 1.** Frequency of antibiotic active ingredients reported by participants (*n*=51). Responses were grouped by active ingredient, aggregating all brand or product names referring to the same compound

Active ingredient	Product name as reported	No. of responses	% of total responses	% of responses by active ingredient
Oxytetracycline	Adamycin	37	73	86
	Alamycin	5	10	
	Tetramycin	1	2	
	Oxytetracycline	1	2	
Penicillin	Penicillin	19	37	43
	Penistrip	3	6	
Tetracycline	Tetracycline	6	12	10
Tylosin	Tylosin	4	8	10
	Tylomix	1	2	
Amoxicillin	Betamox	1	2	2
Tobramycin	Toromycin	1	2	2

In addition to antibiotic use, a high prevalence of anthelmintic drug administration was observed, with 69% (*n*=35) of respondents routinely using these drugs. Worryingly, many farmers conflated anthelmintics with antibiotics, highlighting a lack of understanding regarding the distinction between these two classes of drugs.

### Duration and adherence to treatment

There was no clear consensus amongst participants regarding when to cease administering antibiotics. Half of the respondents (50%) indicated that they stopped treatment once the animal’s symptoms had subsided, even if this required multiple doses (12%, *n*=6). Only a small proportion (4%, *n*=2) reported completing the full course of antibiotics as directed. A further 12% (*n*=6) stated that they stopped treatment based on a veterinarian’s advice, and 4% (*n*=2) followed the instructions provided on the antibiotic packaging or leaflet.

### Sources of advice and antibiotics

**Fig. 2.**
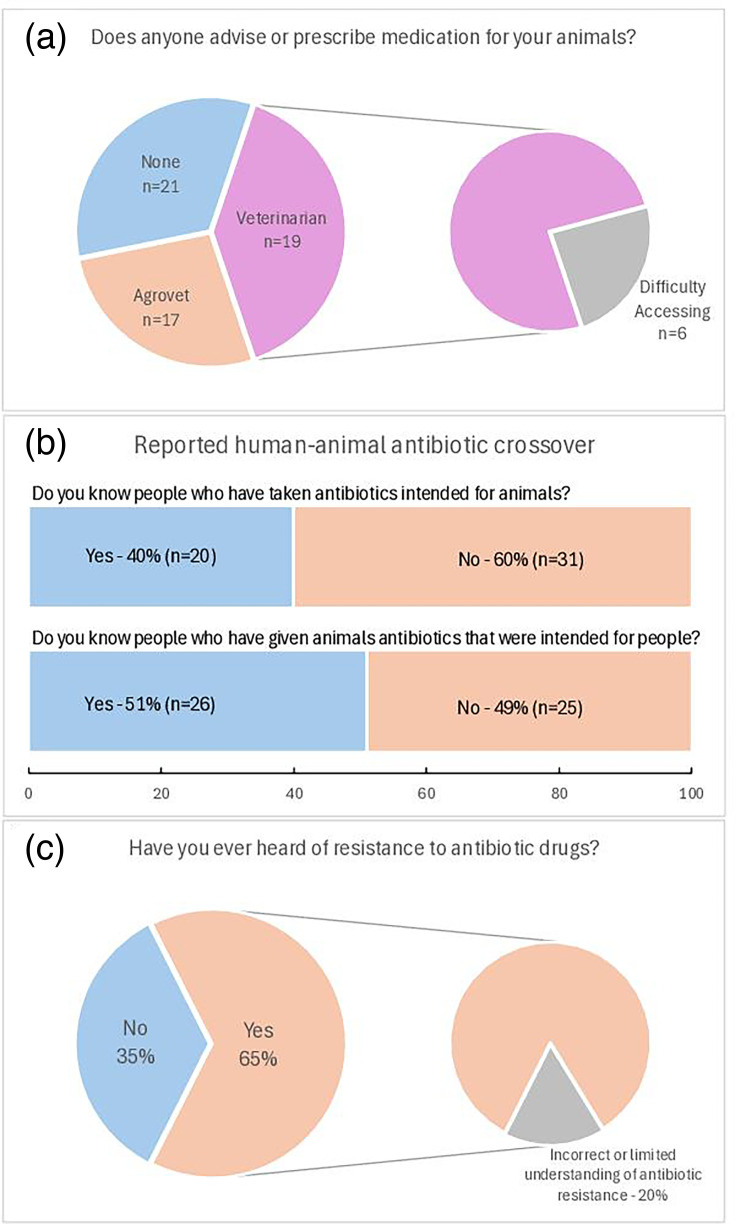
Perceptions and practices surrounding antibiotic use and advice-seeking. (**a**) Sources of advice or prescriptions for animal prescriptions reported by participants. (**b**) Reported instances of human–animal antibiotic crossover. (**c**) Proportion of respondents who have heard of antibiotic resistance and their level of understanding.

Advice on antibiotic use was most often sought from veterinarians (37%, *n*=19), although 12% (*n*=6) of these respondents noted the difficulty of accessing veterinary services, which were sometimes available only annually (Fig. 2a). In this context, participants used the term ‘veterinarian’ specifically to refer to government-employed, legally recognized veterinary officers, distinct from other community-level or informal animal health providers.

Agrovet suppliers were the source of advice for 33% (*n*=17) of participants, whilst 41% (*n*=21) of farmers reported that they did not seek professional guidance (Fig. 2a). Instead, these farmers relied on informal sources, such as family (one respondent), neighbours (two respondents) or their own experience. Similarly, most participants obtained antibiotics from agrovets (94%, *n*=48), whilst a quarter (25%, *n*=13) reported acquiring antibiotics from general shops, with some overlap between these sources.

### Human–animal antibiotic crossover

An area of significant concern was the crossover between human and veterinary antibiotic use. Over half of the participants (51%) reported using animal antibiotics (Fig. 2b), particularly oxytetracycline (14%, *n*=7), in humans, sometimes mixing them into milk or tea. Conversely, 40% (*n*=20) of farmers admitted to using human antibiotics, such as amoxicillin, for treating livestock (Fig. 2b), with 16% (*n*=8) reporting the use of such antibiotics in chickens. Furthermore, 61% (*n*=31) of participants reported consuming meat or milk immediately after administering antibiotics to livestock, with the remaining farmers waiting up to 96 h, raising concerns about antibiotic residue in the food supply chain.

### Knowledge of AMR

The data revealed limited awareness of AMR amongst the farmers.

When asked, ‘Have you ever heard of resistance to antibiotic drugs?’, 40% (*n*=20) of respondents indicated that they had never heard of the concept (Fig. 2c). Amongst those who were familiar with the term, 12% (*n*=6) described resistance in terms of the drug no longer curing animals as it had previously or requiring a different antibiotic to achieve the same effect. These responses suggest a common lay interpretation of AMR as antibiotic ineffectiveness, focusing on the outcome of treatment failure rather than recognizing the biological mechanism whereby bacteria become resistant.

Additionally, 92% (*n*=47) of participants reported storing leftover antibiotics for future use, with all of these farmers using leftover doses on other animals. Some 67% (*n*=34) retained antibiotics until they expired, whilst 8% (*n*=4) used them until they were finished, and the remainder used them intermittently. Sharing antibiotics with neighbours occurred in 4% (*n*=2) of cases.

### Summary and implications

These findings underscore a fluid and inconsistent approach to antibiotic use amongst rural Kenyan livestock farmers, with a significant overlap in human and veterinary antibiotic use, potentially exacerbating the risk of antibiotic resistance. Additionally, the widespread confusion between antibiotics and anthelmintics, as well as the inconsistent adherence to dosing regimens, highlights an urgent need for targeted education and veterinary support to mitigate the risks associated with inappropriate antibiotic use.

## Discussion

### Knowledge gaps and misuse of antibiotics in livestock

AMR is a critically important issue within the One Health framework [23]; addressing it requires a comprehensive approach that includes managing antimicrobial use across all sectors, especially in agriculture, where misuse is prevalent. This research reveals significant knowledge gaps in agropastoral communities in rural Kenya, particularly in understanding antibiotics – specifically, what they are and how and when they should be used. This lack of understanding has led to widespread misuse and inappropriate practices. Additionally, limited awareness of AMR was observed, likely influenced by restricted access to veterinary support.

Throughout our interviews, most participants reported using antibiotics when their animals appeared unwell. This routine use of antibiotics, without an appropriate assessment to consider other causes of illness, such as viral or fungal causes, again leads to an increased risk of resistance [24]. In these situations, veterinary support is crucial for accurate diagnosis; however, it has been estimated that the ratio of veterinarians to livestock in Kenya is 20 times lower than in high-income countries, such as France or Spain [25].

### Barriers to veterinary support and professional advice

Throughout our interviews, the lack of veterinary services was clear, with corresponding reduced access to professional advice, antibiotic prescriptions and vaccination. This means that pastoralists often rely on their neighbours or local agrovets for advice, where antimicrobial use is typically not based on evidence-based practices [19,21,25]. Instead, recommendations for antimicrobials are often influenced by cost and availability [20,25]. If they need additional help, pastoralists often have to rely on marketing materials and informational leaflets that come with the antibiotic packaging. This can be particularly worrisome, as some antibiotics are directly marketed for livestock growth promotion [26]. Indeed, in our interviews, some participants reported administering antibiotics to otherwise healthy animals in order to increase their weight or milk production. This has been shown to be another contributing factor to the increase in global AMR [24].

### Risks associated with informal antibiotic procurement

It is also important to consider the potential quality of medications obtained through informal or unregulated sources, as highlighted in other studies. Counterfeit medications, including antibiotics and antimalarials, are prevalent in many regions, particularly in LMICs [27]. In the context of our findings, farmers relying on agrovets or general shops may face risks associated with substandard or falsified antibiotics, which can compromise treatment efficacy and contribute to AMR.

### Human health implications and food safety concerns

Human health risks are also directly impacted. For example, a review of bacterial isolates from the Isiolo region showed that widespread antibiotic use in cattle herds led to the presence of multi-drug-resistant *Staphylococcus aureus* in raw milk [28]. The consumption of raw milk remains widespread, especially in pastoralist communities, which increases the risk of resistant infectious diseases in human populations. Indeed, in our interviews, many participants reported consuming meat and milk immediately or shortly after administering antibiotics to their animals.

### Awareness of AMR and opportunities for community engagement

Unfortunately, understanding of AMR was limited, with most participants confusing it with treatment failure, consistent with findings from similar research in the region [20,29]. Interestingly, some were aware of AMR in relation to tuberculosis (TB), likely due to public health campaigns highlighting the risk of multidrug-resistant TB in humans. This awareness is promising and highlights the need for similar campaigns focused on animal health. Going forward, we are using the results of these surveys to inform the training of community health promoters and to develop a series of educational workshops focused on AMR [30]. These workshops will aim to bridge knowledge gaps and promote evidence-based practices within the community.

### Limitations

This study was conducted in a small, geographically isolated region of northern Kenya, which may limit the transferability of findings to other pastoralist communities. Data collection coincided with a period of environmental extremes, transitioning from a prolonged 5-year drought to unusually heavy rains, which affected livestock numbers and limited participant availability. Additionally, the reliance on self-reported data may introduce recall or social desirability bias, particularly regarding antibiotic use and practices around human–animal medicine crossover.

## Abbreviations

AMR: antimicrobial resistance
LMICs: low- and middle-income countries
SDGs: Sustainable Development Goals
TB: tuberculosis
WHO: World Health Organization

## References

[1] Dillon M, Kubai E, Doig I, Beattie M (2024).

[2] O’Neill J (2016). Tackling drug-resistant infections globally: final report and recommendations. https://wellcomecollection.org/works/thvwsuba.

[3] Van Boeckel TP, Brower C, Gilbert M, Grenfell BT, Levin SA (2015). Global trends in antimicrobial use in food animals. Proc Natl Acad Sci USA.

[4] World Health Organization (WHO) (2016). Global action plan on antimicrobial resistance. https://www.who.int/publications/i/item/9789241509763.

[5] Kimera ZI, Mshana SE, Rweyemamu MM, Mboera LEG, Matee MIN (2020). Antimicrobial use and resistance in food-producing animals and the environment: an African perspective. Antimicrob Resist Infect Control.

[6] Fratkin E, Roth EA

[7] Homewood K, Kristjanson P, Trench PC Staying Maasai.

[8] United Nations (2015). Transforming our world: the 2030 agenda for sustainable development. https://sdgs.un.org/2030agenda.

[9] Galvin KA (2009). Transitions: pastoralists living with change. Annu Rev Anthropol.

[10] Mburu CM, Bukachi S, Majiwa H, Ongore D, Baylis M (2023). Prioritization of livestock diseases by pastoralists in Oloitoktok Sub County, Kajiado County, Kenya. PLoS One.

[11] Mangesho PE, Caudell MA, Mwakapeje ER, Ole-Neselle M, Kabali E (2021). “We are doctors”: drivers of animal health practices among Maasai pastoralists and implications for antimicrobial use and antimicrobial resistance. Prev Vet Med.

[12] Mangesho PE, Caudell MA, Mwakapeje ER, Ole-Neselle M, Kimani T (2021). Knowing is not enough: a mixed-methods study of antimicrobial resistance knowledge, attitudes, and practices among Maasai pastoralists. Front Vet Sci.

[13] Caudell MA, Quinlan MB, Subbiah M, Call DR, Roulette CJ (2017). Antimicrobial use and veterinary care among agro-pastoralists in northern Tanzania. PLoS One.

[14] Robinson TP, Bu DP, Carrique-Mas J, Fèvre EM, Gilbert M (2017). Antibiotic resistance: mitigation opportunities in livestock sector development. Animal.

[15] Omulo S, Thumbi SM, Njenga MK, Call DR (2015). A review of 40 years of enteric antimicrobial resistance research in Eastern Africa: what can be done better?. Antimicrob Resist Infect Control.

[16] Arsène MMJ, Davares AKL, Viktorovna PI, Andreevna SL, Sarra S (2022). The public health issue of antibiotic residues in food and feed: causes, consequences, and potential solutions. Vet World.

[17] Katz SE, Brady MS (2000). Antibiotic residues in food and their significance. Food Biotechnology.

[18] Khachatourians GG Agricultural use of antibiotics and the evolution and transfer of antibiotic-resistant bacteria.

[19] Afakye K, Kiambi S, Koka E, Kabali E, Dorado-Garcia A (2020). The impacts of animal health service providers on antimicrobial use attitudes and practices: an examination of poultry layer farmers in Ghana and Kenya. Antibiotics.

[20] Kemp SA, Pinchbeck GL, Fèvre EM, Williams NJ (2021). A cross-sectional survey of the knowledge, attitudes, and practices of antimicrobial users and providers in an area of high-density livestock-human population in Western Kenya. Front Vet Sci.

[21] Kiambi S, Mwanza R, Sirma A, Czerniak C, Kimani T (2021). Understanding antimicrobial use contexts in the poultry sector: challenges for small-scale layer farms in Kenya. Antibiotics.

[22] Braun V, Clarke V (2019). Reflecting on reflexive thematic analysis. Qual Res Sport Exerc Health.

[23] World Health Organization (WHO) (2025). One Health. https://www.who.int/news-room/fact-sheets/detail/one-health.

[24] Manishimwe R, Nishimwe K, Ojok L (2017). Assessment of antibiotic use in farm animals in Rwanda. Trop Anim Health Prod.

[25] Caudell MA, Dorado-Garcia A, Eckford S, Creese C, Byarugaba DK (2020). Towards a bottom-up understanding of antimicrobial use and resistance on the farm: a knowledge, attitudes, and practices survey across livestock systems in five African countries. PLoS One.

[26] McKernan C, Benson T, Farrell S, Dean M (2021). Antimicrobial use in agriculture: critical review of the factors influencing behaviour. *JAC Antimicrob Resist*.

[27] World Health Organization (2024). Substandard and falsified medical products. https://www.who.int/news-room/fact-sheets/detail/substandard-and-falsified-medical-products.

[28] Nayiga S, Kayendeke M, Nabirye C, Willis LD, Chandler CIR (2020). Use of antibiotics to treat humans and animals in Uganda: a cross-sectional survey of households and farmers in rural, urban and peri-urban settings. *JAC Antimicrob Resist*.

[29] Shitandi A, Sternesjö A (2004). Factors contributing to the occurrence of antimicrobial drug residues in Kenyan milk. J Food Prot.

[30] Lemma M, Alemu B, Mekonnen M, Wieland B (2019). Community conversations on antimicrobial use and resistance. https://cgspace.cgiar.org/items/b91585ac-e795-4809-a60f-63f66b774c02.

